# Adherence to antiretroviral therapy for HIV in sub-Saharan Africa and Asia: a comparative analysis of two regional cohorts

**DOI:** 10.7448/IAS.20.1.21218

**Published:** 2017-03-03

**Authors:** Rimke Bijker, Awachana Jiamsakul, Cissy Kityo, Sasisopin Kiertiburanakul, Margaret Siwale, Praphan Phanuphak, Sulaimon Akanmu, Romanee Chaiwarith, Ferdinand W Wit, Benedict LH Sim, Tamara Sonia Boender, Rossana Ditangco, Tobias F Rinke De Wit, Annette H Sohn, Raph L Hamers

**Affiliations:** ^a^ Amsterdam Institute for Global Health and Development and Department of Global Health, Academic Medical Center of the University of Amsterdam, Amsterdam, The Netherlands; ^b^ The Kirby Institute, Biostatistics and Databases Program, UNSW Australia, Sydney, NSW, Australia; ^c^ Joint Clinical Research Centre, Kampala, Uganda; ^d^ Faculty of Medicine, Ramathibodi Hospital, Mahidol University, Bangkok, Thailand; ^e^ Lusaka Trust Hospital, Lusaka, Zambia; ^f^ HIV-NAT/Thai Red Cross AIDS Research Centre, Bangkok, Thailand; ^g^ College of Medicine, University of Lagos, Lagos, Nigeria; ^h^ Division of Infectious Diseases, Department of Medicine, Research Institute for Health Sciences, Chiang Mai, Thailand; ^i^ Infectious Disease Unit, Department of Medicine, Hospital Sungai Buloh, Sungai Buloh, Malaysia; ^j^ Research Institute for Tropical Medicine, Manila, Philippines; ^k^ TREAT Asia/amfAR, The Foundation for AIDS Research, Bangkok, Thailand; ^l^ Department of Internal Medicine, Division of Infectious Diseases, Academic Medical Center of the University of Amsterdam, Amsterdam, The Netherlands

**Keywords:** HIV-1, antiretroviral therapy (ART), adherence, Asia, sub-Saharan Africa

## Abstract

**Introduction**: Our understanding of how to achieve optimal long-term adherence to antiretroviral therapy (ART) in settings where the burden of HIV disease is highest remains limited. We compared levels and determinants of adherence over time between HIV-positive persons receiving ART who were enrolled in a bi-regional cohort in sub-Saharan Africa and Asia.

**Methods**: This multicentre prospective study of adults starting first-line ART assessed patient-reported adherence at follow-up clinic visits using a 30-day visual analogue scale. Determinants of suboptimal adherence (<95%) were assessed for six-month intervals, using generalized estimating equations multivariable logistic regression with multiple imputations. Region of residence (Africa vs. Asia) was assessed as a potential effect modifier.

**Results**: Of 13,001 adherence assessments in 3934 participants during the first 24 months of ART, 6.4% (837) were suboptimal, with 7.3% (619/8484) in the African cohort versus 4.8% (218/4517) in the Asian cohort (*p* < 0.001). In the African cohort, determinants of suboptimal adherence were male sex (odds ratio (OR) 1.27, 95% confidence interval (CI) 1.06–1.53; *p* = 0.009), younger age (OR 0.8 per 10 year increase; 0.8–0.9; *p* = 0.003), use of concomitant medication (OR 1.8, 1.0–3.2; *p* = 0.044) and attending a public facility (OR 1.3, 95% CI 1.1–1.7; *p* = 0.004). In the Asian cohort, adherence was higher in men who have sex with men (OR for suboptimal adherence 0.6, 95% CI 0.4–0.9; *p* = 0.029) and lower in injecting drug users (OR for suboptimal adherence 1.6, 95% CI 0.9–2.6; *p* = 0.075), compared to heterosexuals. Risk of suboptimal adherence decreased with longer ART duration in both regions. Participants in low- and lower-middle-income countries had a higher risk of suboptimal adherence (OR 1.6, 1.3–2.0; *p* < 0.001), compared to those in upper-middle or high-income countries. Suboptimal adherence was strongly associated with virological failure, in Africa (OR 5.8, 95% CI 4.3–7.7; *p* < 0.001) and Asia (OR 9.0, 95% CI 5.0–16.2; *p* < 0.001). Patient-reported adherence barriers among African participants included scheduling demands, drug stockouts, forgetfulness, sickness or adverse events, stigma or depression, regimen complexity and pill burden.

**Conclusions**: Psychosocial factors and health system resources may explain regional differences. Adherence-enhancing interventions should address patient-reported barriers tailored to local settings, prioritizing the first years of ART.

## Introduction

The introduction of combination antiretroviral therapy (ART) has dramatically reduced morbidity, mortality and infectiousness of persons infected with HIV [1]. Of the estimated 17 million people receiving ART worldwide, about 12 million reside in sub-Saharan Africa and 2 million in Asia, the two regions most affected by the HIV epidemic [1].

Consistently high levels of adherence to ART are essential for sustained viral suppression, thus preventing drug resistance [2] and disease progression [3]. Reported correlates of adherence include patient-related factors (such as understanding the utility of ART and adherence, trust in the care provider, HIV-associated stigma), treatment-related factors (such as regimen complexity), psychosocial factors (such as depressive symptoms) and socioeconomic factors (such as financial constraints) [4,5]. These factors can differ greatly across regions and populations, due to variations in sociodemographics, culture and availability of resources.

The Joint United Nations Programme on HIV/AIDS (UNAIDS) 90–90–90 global treatment targets emphasize sustained viral suppression and therewith the need for optimal adherence [6]. Characterizing and understanding determinants of adherence in settings with the highest disease burden will be critical to attaining these targets. Despite favourable short-term data from low-resource settings [7–10], concerns remain that long-term adherence may be suboptimal because of multiple barriers including lack of basic health education and enrolment in massive ART programmes with limited capacity for patient monitoring and support.

Two meta-analyses identified a number of correlates/predictors and patient-reported barriers of adherence [4,5], but were hampered by large heterogeneity across studies in adherence method and study design [4] and limited data from developing world settings [5].

We, therefore, conducted a prospective analysis of ART adherence among HIV-positive adults receiving first-line ART in sub-Saharan Africa and Asia, who were enrolled in a bi-regional monitoring programme [11]. The use of a generic study protocol and standard self-report adherence measure allowed for a substantive assessment and comparison of the levels and determinants of long-term adherence between populations residing in culturally and economically diverse regions with the highest burden of HIV disease

## Methods

### Study design and population

The PanAfrican (PASER-M) and TREAT Asia (TASER-M) Studies to Evaluate Resistance-Monitoring are two parallel regional cohorts of HIV-1 infected adults (aged ≥18 years) receiving care at 13 clinical sites in 6 African countries (Kenya, Nigeria, South Africa, Uganda, Zambia and Zimbabwe) and 12 clinical sites in 5 Asian countries (Hong Kong SAR, Indonesia, Malaysia, Philippines and Thailand), sharing a generic study protocol, the same patient eligibility criteria and standard adherence measure, as previously profiled [11].

The present study included all cohort participants who initiated first-line ART and were followed up (time period: 2007–2013) in accordance with national guidelines, because of advanced immunodeficiency (defined by a CD4 cell count <200 or <350 cells/μl) or clinical disease (according to WHO or CDC classification) [12]. Previous use of any antiretroviral drugs to prevent mother-to-child transmission of HIV was not an exclusion criterion. We excluded persons who did not have at least one documented adherence assessment during the first 24 months on ART, or who were not retained in care for at least three months after ART initiation.

Participants provided written informed consent at enrolment. The study protocol was approved by the appropriate national and local research ethics committees at all collaborating sites.

### Data collection and outcome measure

Participants received care in accordance with the local standard-of-care guidelines. Personnel at all sites had received training in adherence assessment and counselling. Self-reported drug adherence in the past 30 days was assessed at each follow-up clinic visit using the visual analogue scale (range 0–100%) [13,14]. Adherence outcomes were defined for six-month intervals (6, 12, 18, 24 months after ART initiation), allowing six-month time windows (3–9, 9–15, 15–21 and 21–27 months, respectively). If more than one adherence assessment was performed within a six-month interval, the mean was used. Adherence was classified as optimal (≥95%) or suboptimal (<95%) for each six-month interval [15–17]. For descriptive purposes, adherence levels were additionally categorized as <80, 80–94, 95–99 and 100%.

Follow-up time was measured from ART initiation and ended at the earliest of either last follow-up visit or 24 months after starting ART. The analysis was censored at time of death, transfer out, ART discontinuation, switch to second-line therapy because of treatment failure, or loss to follow-up.

Other collected data included pre-ART demographics, clinical information at each follow-up clinic visit, and annual plasma HIV viral load. Among African participants, patient-reported adherence barriers were recorded among those who reported missed pills using a standardized questionnaire.

### Statistical analysis

We fit a multivariable logistic regression model to estimate the associations between several covariates and suboptimal adherence. Generalized estimating equations were used to account for the correlation of repeated measurements within participants [18], specifying a first-order auto-regressive variance-covariance correlation structure. The quasi-likelihood information criterion statistic was used to evaluate fit. To account for missing data, we did multiple imputations for independent variables (pre-ART viral load, CD4 cell count, concomitant medication; 11.7% of timepoints) and adherence (17.4% of timepoints), using the Markov chain Monte Carlo approach with 20 simulation datasets. We tested whether the missing data were missing at random (or at least approximately so), and concluded that use of multiple imputations was justified (Supplementary Table S1).

#### Main analysis

The main analysis was a model that incorporated the following a priori chosen variables: sex, age (continuous, per 10-year increase), HIV risk group (heterosexual contact, men who have sex with men (MSM), injecting drug use (IDU) or unspecified), pre-ART history of AIDS, pre-ART CD4 cell count (<50, 50–99, 100–199, ≥200 cells/μl), pre-ART viral load (<10,000, 10,000–99,999 or ≥100,000 copies/ml), type of ART regimen (non-nucleoside reverse transcriptase inhibitor (NNRTI) based or other), most recent CD4 cell count (continuous, per 100 cells/μl increase), use of any concomitant medication, public versus non-public sector health facility, and time on ART (6, 12, 18 or 24 months). If more than one measurement of CD4 cell count was available within a six-month interval, the mean was used. Because univariate analysis showed significant interaction between region and multiple independent variables, the main analysis was stratified for the African and Asian cohorts. All analyses were adjusted for calendar year of ART initiation, number of adherence assessments, and clinic type (general hospital, HIV clinic, or teaching hospital).

#### Additional analyses

(1) We examined the association between suboptimal adherence and virological failure (plasma viral load ≥400 copies/ml) using multivariable logistic regression, for the 12 and 24 months intervals and for each region. (2) To assess the association between long-term ART duration and adherence, we repeated the analysis using the data of the 15 of 25 sites (5/13 in Africa and 10/11 in Asia) that had followed participants up to 36 months. (3) To assess the association between specific antiretroviral drugs and adherence, we repeated the analysis including only the participants on an NNRT-based regimen and adding two additional variables: type of nucleoside reverse transcriptase inhibitor (NRTI) backbone (containing zidovudine, tenofovir, stavudine or other) and type of NNRTI (efavirenz or nevirapine). (4) To investigate whether differences in adherence were attributable to resources available to the population at large, we repeated the analysis with the two regional cohorts aggregated into one model including a binary variable for country income status: low or lower-middle income (Indonesia, Kenya, Nigeria, Philippines, Uganda, Zambia and Zimbabwe) versus upper-middle or high income (Hong Kong SAR, Malaysia, South Africa, Thailand), based on 2013 World Bank definitions [19].

#### Sensitivity analyses

(1) To assess the robustness of our results, we modified the definition of the outcome measure, either using different cutoffs (<90% or <100%) to define suboptimal adherence, or using the lowest measured adherence level for each six-month interval (if >1 assessment was available) with a <95% cutoff – instead of mean adherence. (2) It could be argued that improving adherence with longer ART duration could be due to attrition (lost to follow-up, ART discontinuation, death or regimen switch). To investigate possible attrition bias, we performed a sensitivity analysis in which follow-up time for all participants was extended to 24 months regardless of their status, while imputing missing adherence assessments and time-changeable variables using last observation carried forward (LOCF) methods. Adherence was also filled backwards to earlier time intervals, if missing, using the first recorded data.

Results were expressed using odds ratios (ORs) with 95% confidence intervals (CIs) and two-sided *p*-values (*p* < 0.05 significant). All statistical analyses were performed with Stata version 12 (StataCorp LP, TX, USA).

## Results

### Study population

Of 4659 participants starting first-line ART, we excluded 430 (9%) because they had less than three months follow-up time and 295 (6%) because they did not have at least one recorded adherence assessments during 24 months of follow-up. The analysis included 3934 participants, 2424 (62%) from Africa and 1510 (38%) from Asia (Table 1). Countries of residence were Uganda (24%), South Africa (23%), Zambia (20%), Kenya (17%), Zimbabwe (9%), and Nigeria (7%) for Africa, and Thailand (57%), Malaysia (21%), Hong Kong SAR (11%), Philippines (9%) and Indonesia (2%) for Asia. 2030 (52%) participants came from low- or lower-middle-income countries, and 1904 (48%) came from upper-middle- or high-income countries. Most African participants were women (59%, 1441/2424), and most Asians were men (67%, 1008/1510). Advanced HIV before ART start was less frequent among the African than the Asian participants, with CD4 cell counts <100 cells/μl in 35% and 45%, respectively, and a history of AIDS in 15% and 51%, respectively. NNRTI-based regimens were predominant in both regions. All sites in Asia and 6 of 13 sites in Africa were public sector facilities.

**Table 1.** Participant characteristics at ART initiation.

	Total	Africa	Asia	
	*n* = 3934	*n* = 2424	*n* = 1510	*p*-Value
Sex				<0.001
Female	1943 (49.4)	1441 (59.4)	502 (33.2)	
Male	1991 (50.6)	983 (40.6)	1008 (66.8)	
Age (mean, SD)	37.8 (9.3)	37.8 (9.0)	37.8 (9.8)	0.885
HIV risk group				<0.001
Heterosexual	2637 (67.0)	1631 (67.3)	1006 (66.6)	
MSM	392 (10.0)	4 (0.2)	388 (25.7)	
IDU	87 (2.2)	0 (0.0)	87 (5.8)	
Unspecified	818 (20.8)	789 (32.5)	29 (1.9)	
Previous antiretroviral use				0.825
None	3856 (98.0)	2375 (98.0)	1481 (98.1)	
PMTCT	78 (2.0)	49 (2.0)	29 (1.9)	
History of AIDS				<0.001
No	2801 (71.2)	2054 (84.7)	747 (49.5)	
Yes	1133 (28.8)	370 (15.3)	763 (50.5)	
Pre-ART CD4 cell count (median cells/µl, SD)^a^	134 (55–210)	141 (68–209)	119 (40–215)	0.027
<50	915 (23.5)	467 (19.4)	448 (30.3)	<0.001
50–99	607 (15.6)	387 (16.0)	220 (14.9)	
100–199	1238 (31.8)	857 (35.5)	381 (25.7)	
200–349	1061 (29.2)	680 (28.2)	381 (25.7)	
≥350	73 (1.9)	23 (0.9)	50 (3.4)	
Pre-ART HIV viral load (median log_10_ cps/ml, IQR)^b^	5.0 (4.4–5.5)	5.0 (4.3–5.6)	5.0 (4.5–5.4)	0.668
ART regimen				<0.001
NNRTI-based	3822 (97.2)	2418 (99.8)	1404 (93.0)	
Other^c^	112 (2.8)	6 (0.2)	106 (7.0)	
Calendar year of ART initiation				<0.001
2007	706 (18.0)	565 (23.3)	141 (9.3)	
2008	2000 (50.8)	1499 (61.8)	501 (33.2)	
2009	879 (22.3)	360 (14.9)	519 (34.4)	
2010	349 (8.9)	–	349 (23.1)	
Sector				<0.001
Non-public	1296 (32.9)	1296 (53.5)	–	
Public	2638 (67.1)	1128 (46.5)	1510 (100.0)	
Country income status				<0.001
Low/lower-middle income	2030 (51.6)	1873 (77.3)	157 (10.4)	
Upper-middle/high income	1904 (48.4)	551 (22.7)	1353 (89.6)	

ART, antiretroviral therapy; SD, standard deviation; IQR, interquartile range; MSM, men who have sex with men; IDU, injecting drug use; PMTCT, prevention of mother-to-child-transmission; NNRTI, non-nucleoside reverse transcriptase inhibitor.

All data are *n* (%), unless indicated otherwise.

^a^Data available for 3894 participants.

^b^Data available for 3890 participants.

^c^87 participants on protease inhibitor regimens and 25 participants on triple nucleoside reverse transcriptase inhibitor regimens.

### Description of outcomes

After 24 months of follow-up, 3223 (82%) of 3934 participants were alive and retained on first-line ART; 80 (2%) died, 112 (3%) transferred out, 32 (0.8%) discontinued ART, 315 (8%) were lost to follow-up, and 172 (4%) were switched to second-line therapy (Supplementary Figure S1 and S2). Participant retention was similar in the African (81%, 1972/2424) and Asian cohorts (83%, 1251/1510). The proportions of participants with viral suppression (<400 copies/ml) at 12, 24 months and overall were 90% (1867/2073), 90% (1608/1792) and 90% (3475/3865), respectively, in the African cohort, and 94% (1072/1143), 96% (887/922) and 95% (1959/2065), respectively, in the Asian cohort (Africa vs. Asia, *p* < 0.001).

A total of 23,278 adherence assessments were performed, with a median number per participant of 7 (interquartile range (IQR) 6–7) in Africans and 8 (IQR 5–9) in Asians (Table 2). The proportion of suboptimal adherence was 6.4% (837/13,001) overall, 7.3% (619/8484) in the African cohort and 4.8% (218/4517) in the Asian cohort (*p* < 0.001) (Table 2). This regional difference was noted for each time point: 10.2% versus 7.5% for month 6, 7.8% versus 5.6% for month 12, 5.5% versus 3.6% for month 18, and 5.1% versus 2.3% for month 24, respectively.

**Table 2.** Summary of adherence assessments.

	Total		Africa		Asia	
	*n*	(%)	*n*	(%)	*n*	(%)
Participants	3913	100	2424	61.6	1510	38.4
Adherence assessments	23,278	100	14,714	63.2	8564	36.8
Median (IQR) number of adherence assessments per participant	7	6–8	7	6–7	8	5–9
Mean adherence assessments	13,001	100	8484	65.3	4517	34.7
Suboptimal adherence	837	6.4	619	7.3	218	4.8

IQR, interquartile range.

Data are *n* (%) unless indicated otherwise.

Adherence assessments used the 30-day self-reported visual analogue scale.

Mean adherence was calculated if >1 adherence assessment was performed within a time interval, and classified as optimal (≥95%) or suboptimal (<95%).

**Figure 1.**
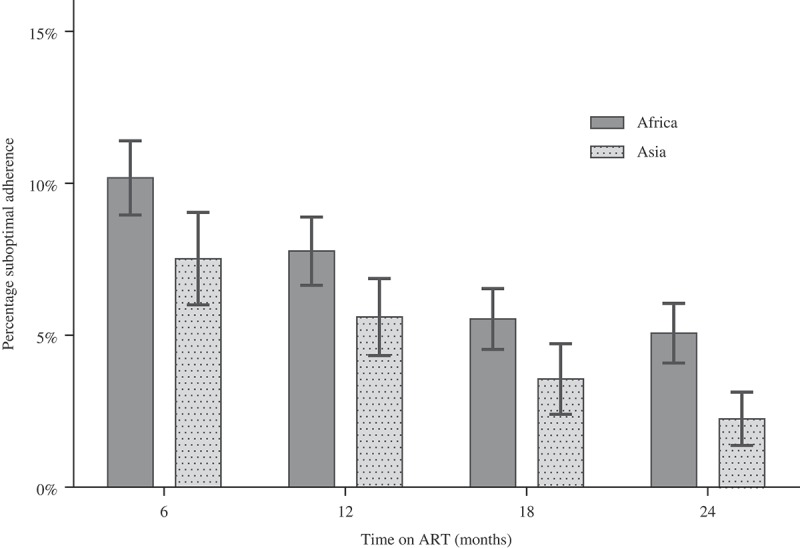
Proportions of suboptimal adherence over time in the African and Asian cohorts. Suboptimal adherence defined as a visual analog score of <95%. ART, antiretroviral therapy.

**Figure 2.**
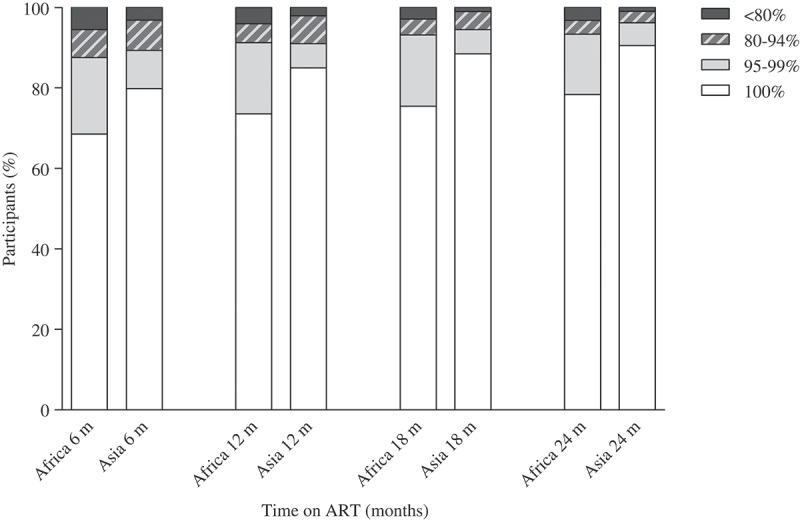
Categories of adherence levels over time in the African and Asian cohorts. Vertical bars represent 95% confidence intervals.

The proportion of suboptimal adherence decreased over time, with relative reductions of 50% in the African cohort (from 10.2% to 5.1%) and 69% in the Asian cohort (from 7.5% to 2.3%) (Figure 1). Participants who reported adherence <80% were more frequent in Africa across all time points, compared with Asia. Conversely, participants with perfect (100%) adherence were more frequent in Asia across all time points, compared with Africa (Figure 2).

### Patient-reported adherence barriers

Among the African participants, a total of 235 participants recorded to have missed one or more pills at 1045 clinic visits. The most frequently reported reasons were scheduling demands (such as conflicting work schedule or travel; 33%, 350), followed by pharmacy stockouts (30%, 317), forgetfulness (28%, 297), sickness or adverse events (13%, 135), psychosocial issues (such as stigma and depression; 8%, 88), regimen complexity (4%, 44), pill burden (3%, 35), and other (13%, 134); 35% (82) of participants reported two or more reasons.

### Determinants of adherence

#### Main analysis

Table 3 shows the multivariable regression models stratified by region. In the African cohort, male sex (OR 1.3, CI 1.1–1.5; *p* = 0.009), younger age (OR 0.8 per 10-year increase, CI 0.8–0.9; *p* = 0.003), any concomitant medication (OR 1.8, CI 1.0–3.2; *p* = 0.044), and attending a public facility (OR 1.4, CI 1.1–1.7, *p* = 0.004) were associated with suboptimal adherence. In the Asian cohort, compared to heterosexual exposure, MSM had a lower risk of suboptimal adherence (OR 0.6, CI 0.4–0.9; *p* = 0.029), and IDUs had a higher risk, but this did not reach statistical significance (OR 1.6, CI 1.0–2.6; *p* = 0.075). In both regions, the risk of suboptimal adherence significantly decreased with longer ART duration. Compared to the six-month time interval, participants in both cohorts were less likely to have suboptimal adherence at month 12, 18 and 24 (OR 0.8, 0.6 and 0.5, respectively, in Africa and OR 0.8, 0.6 and 0.4, respectively, in Asia). A higher recent CD4 cell count was associated with better adherence in both regions. Pre-ART HIV viral load, AIDS and CD4 cell count and the type of ART regimen were not associated.

**Table 3.** Factors associated with suboptimal adherence, by region.

	Africa			Asia		
	OR	95% CI	*p*	OR	95% CI	*p*
Sex						
Female	1.00			1.00		
Male	1.27	1.06–1.53	0.009	1.12	0.78–1.61	0.537
Age						
Per 10-year increase	0.85	0.76–0.95	0.003	0.90	0.76–1.07	0.239
HIV risk group						
Heterosexual	1.00			1.00		
MSM ^a^				0.59	0.36–0.94	0.029
IDU				1.58	0.95–2.60	0.075
Unspecified	0.76	0.70–0.82	<0.001	0.40	0.08–1.95	0.258
History of AIDS						
No	1.00			1.00		
Yes	0.97	0.76–1.24	0.816	0.85	0.60–1.21	0.371
Most recent CD4 cell count						
Per 100 cells/µl increase	0.89	0.84–0.95	<0.001	0.90	0.82–1.00	0.050
Pre-ART viral load (cps/ml)						
<10,000	1.00			1.00		
10,000–99,999	1.19	0.88–1.61	0.248	0.63	0.38–1.04	0.071
≥100,000	1.15	0.86–1.53	0.355	0.85	0.55–1.34	0.491
ART regimen						
NNRTI-based	1.00			1.00		
Other	1.49	0.31–7.07	0.615	0.31	0.08–1.22	0.093
ART duration						
6 months	1.00			1.00		
12 months	0.76	0.62–0.93	0.009	0.81	0.58–1.14	0.232
18 months	0.56	0.44–0.71	<0.001	0.56	0.38–0.84	0.006
24 months	0.51	0.39–0.65	<0.001	0.38	0.23–0.64	<0.001
Concomitant medication^b^						
No	1.00			1.00		
Yes	1.80	1.02–3.18	0.044	1.12	0.83–1.53	0.459
Sector						
Non-public	1.00			–^c^		
Public	1.39	1.11–1.74	0.004			

MSM, men who have sex with men; IDU, injecting drug use; NNRTI, non-nucleoside reverse transcriptase inhibitor, OR, odds ratio; CI, confidence interval.

This table shows results of multivariable logistic regression with generalized estimating equation and multiple imputations to model the probability that a participant was in the suboptimal adherence group. Therefore, an OR of >1 indicates an increased odds of suboptimal adherence, compared with the odds for the reference group. Models are adjusted for number of assessments, site type and calendar year of ART initiation. Pre-ART CD4 cell count was not associated in univariate analysis.

^a^MSM category in Africa was included in unknown category due to few observations (*n* = 4).

^b^Use of any other medication simultaneously with ART.

^c^All clinical sites in Asia were public facilities.

#### Additional analyses

(1) Suboptimal adherence was strongly associated with virological failure, both at month 12 (OR 6.5, CI 4.7–9.0; *p* < 0.001) and 24 (OR 7.4, CI 4.7–11.5; *p* < 0.001), and both in Africa (OR 5.8, CI 4.3–7.7; *p* < 0.001) and Asia (OR 9.0, CI 5.0–16.2; *p* < 0.001) (Supplementary Table S2). (2) The long-term analysis up to 36 months of follow-up, including 2434 participants from 15 sites, found that the association between longer ART duration and higher adherence was sustained in both regions, supporting the findings from the main analysis. Compared to the six-month time interval, at month 36 participants from both cohorts were less likely to have suboptimal adherence (OR 0.1; CI 0.06–0.2; *p* < 0.001, in Africa and OR 0.2; CI 0.09–0.4; *p* < 0.001, in Asia). (Supplementary Table S3 and Figure S3.) (3) The by-drug analysis found that none of the individual NRTI or NNRTI drugs were significantly associated with adherence in either region (Supplementary Table S4). (4) In the country income analysis participants from a low- or lower-middle income were 1.6 times (CI 1.3–2.0; *p* < 0.001) more likely to have suboptimal adherence than those from an upper-middle- or high-income country.

#### Sensitivity analyses

(1) Varying the definition of suboptimal adherence yielded largely the same associations as found in the main analysis (Supplementary Tables S5, S6, S7). (2) Using LOCF, longer ART duration remained significantly associated with higher adherence in Africa, but not in Asia. Other associations remained largely similar to the main model (Supplementary Table S8).

## Discussion

On the basis of 23,278 assessments in 3934 HIV-positive patients receiving first-line ART in sub-Saharan Africa and Asia, we observed high proportions of adequate self-reported drug adherence across both regions. During the first two years on ART, participants reported optimal adherence at 93% of clinic visits in the African cohort and 95% of follow-up clinic visits in the Asian cohort. This finding is consistent with two meta-analyses [8,9] and post-hoc analyses of two multi-country clinical trials [7,10] that reported favourable levels of adherence in low- and middle-income countries.

The 30-day VAS adherence measure was found to be strongly associated with virological outcomes in both regional cohorts, which corroborates the robustness of the observed associations. It needs to be recognized, however, that some studies have found self-reported adherence measures to be only moderately sensitive in predicting immunological or virological outcomes [20,21]. Recent WHO guidelines recommend viral load as the preferred monitoring strategy to assess ART effectiveness [22].

Despite overall high levels of adherence in the cohorts, we documented suboptimal adherence in a modest but relevant fraction of participants, which is a cause for concern. Participants in Africa reported lower adherence levels compared to their Asian counterparts. Correspondingly, viral suppression rates in the on-treatment populations were lower in the African cohort (90%), compared with the Asian cohort (95%).

We identified several determinants of suboptimal adherence, with some notable differences between the regions. First, a lower income status of the country of residence was found to be an independent risk factor of suboptimal adherence. Better adherence may partly be attributed to the resources available within a given national health system [23–25], or, more specifically, the ART site, for instance in terms of staff training levels, patient-staff ratios and patient monitoring strategies. In this respect, the Asian study sites were generally better resourced compared to their African counterparts. Our finding that in Africa adherence levels were lower in public facilities, which are typically less well-resourced, than non-public facilities, further supports this notion. Second, psychosocial and lifestyle differences between regions may play an important role. For instance, the subset of MSM participants in Asia reported higher adherence than heterosexuals, which expands on earlier reports of high adherence among MSM populations [9,26]. Among African male participants, MSM behaviour may be underrepresented (*n* = 4) due to stigma and associated limited access to HIV services [27]. Third, social desirability bias between the regions may have influenced the observed differences; however, given the corresponding regional differences in viral suppression rates, such an effect is likely to be limited.

Additional findings provide some relevant new insights. In both the African and Asian cohorts, drug adherence improved consistently over time up to at least three years of follow-up. Attrition rates in the early stages of ART in low- and middle-income countries are known to be high, because of early mortality and loss to follow-up [28]. In an attempt to account for potential attrition bias – by use of multiple imputations for missing data as well as a sensitivity analysis using LOCF methods – adherence levels maintained their improvement over time in the African cohort; in Asia, however, improvement over time was no longer statistically significant in the LOCF analysis. These findings indicate that the significant effect of time was not entirely influenced by patient attrition, suggesting that patients who have been on ART for a longer period of time without defaulting may be more likely to be adherent. Patients may have become more adept at taking drugs on a daily basis, early side effects may have disappeared with time, or they may have received targeted adherence counselling and support. Our findings are in line with a UK study with nine years of follow-up that found a consistent increase of 2% per year in adherence over time [29] and a French study with three years of follow-up that found that high early adherence was independently associated with a favourable long-term immunovirological response [30]. Our findings demonstrate that interventions to support adherence and reduce adherence barriers should be a priority in the early stages of ART.

Clarifying the reasons for missing pills can improve our understanding of the multifaceted challenges people encounter in maintaining life-long adherence to ART. The patient-reported barriers to adherence among the African participants varied considerably, and included scheduling demands, forgetfulness, sickness or adverse events, stigma or depression, regimen complexity, and pill burden. This reflects the diversity of the population affected by HIV and highlights the importance of tailoring adherence-support interventions to the individual and their sociocultural context [31,32]. Two recent meta-analyses of studies from sub-Saharan Africa summarized the available evidence of potential interventions that can promote ART adherence, suggesting that mobile phone text messages or other reminder devices, treatment supporters, education and counselling, and providing food supplements can be effective approaches in some settings [32,33]. Nonetheless, general limitations in available data emphasize that there is a need for more research to better examine the influence of sociocultural context and the content of potential adherence-support interventions that are relevant to local settings.

Furthermore, pharmacy drug stockouts were among the most frequently reported reasons for missing pills in Africa, illustrating deficiencies in service delivery rather than individual patients’ lack of adherence. This finding endorses previous evaluations that identified important gaps in many procurement and supply systems of ART programmes in low- and middle-income countries [34]. These programmatic deficiencies need to be urgently addressed to preserve the durable effectiveness of first-line regimens and achieve sustained ART success.

This study found that use of individual NRTIs or NNRTIs were not associated with adherence, and that use of concomitant medication was associated with suboptimal adherence among the African participants. However, it needs to be recognized that this study was not able to fully assess the potential effects of pill burden, single-tablet or once-daily ART regimens [35]. Also, only few patients received a non-NNRTI-based regimen, which precluded a comparison with other regimens (e.g. protease inhibitor-based). Among the African participants, poor adherence was associated with younger age, concurring with several previous studies [7,29], and male sex. By contrast, in Asia, sex or age were not associated. A meta-analysis suggested that overall adherence levels may be higher in men than women, and that such sex differences could be attributable to several factors, such as – among men – being MSM or reporting no alcohol abuse, and – among women – having lower pre-ART CD4 cell counts, being a widow or residing in a low- or middle-income country [26]. Therefore, in the present study, it is likely that additional unmeasured factors contributed to the observed regional differences.

The main strengths of the study were the prospective design with long-term follow-up and repeated adherence measurements, the large number of patients and clinical sites included in the aggregated analysis, and that we were able to correlate the self-report adherence measure with viral load outcomes. The outcome measure was robust in sensitivity analyses. The setting of routine ART programmes with wide geographic coverage enhanced the generalizability of the results, although the study population was not necessarily representative of all people with HIV/AIDS in the regions studied and caution is warranted when extrapolating results to different subpopulations or countries. The use of a common core study protocol with a standard, validated adherence measure across both regions added to the internal validity of our findings.

The study has some limitations. First, there was incomplete data ascertainment for a number of factors that can affect adherence both in terms of patient attributes (such as substance abuse and mental health), regimen characteristics (such as single-tablet regimens), and health-care-related factors (such as differences in health care models and policies to promote retention in care). The self-reported barriers to adherence (i.e. reasons for missed pills) among African participants underline the importance of including such factors in future research. Second, there are various sources of heterogeneity in the study population, both within and between the two regions studied, that could not be completely accounted for in the analysis, for instance differences in health care organization and human behaviour, which could have influenced the findings. Third, our findings mainly applied to patients who are severely immunocompromised (CD4 <200 cells/µl) at ART initiation, limiting generalizability to populations adopting a “test-and-treat” approach (who may be immunocompetent and therefore asymptomatic) in accordance with the latest WHO treatment guidelines [22]. Fourth, because of the observational design of the study, the associations found do not necessarily demonstrate causality. Also, despite our efforts to reduce attrition bias by applying multiple imputations and performing a sensitivity analysis, residual unmeasured confounding could have influenced some of the findings. Lastly, the analysis was limited to adults; ART-treated adolescents and children may face different adherence barriers to those identified in this study.

### Conclusions

Comparing the determinants of suboptimal adherence to ART between the world’s two most HIV-affected regions offered the potential to identify patients at risk, guide targets for developing interventions to enhance or maintain adherence, thus addressing the global challenge of providing effective long-term treatment to millions of patients in Africa and Asia. The study findings emphasized the influence of psychosocial factors and health system resources on ART adherence. Adherence-enhancing interventions need to address patient-reported barriers tailored to local settings, prioritizing the first years of ART. Improved drug procurement and supply systems are required for sustained ART success in low-resource settings.

## Notes

The TREAT Asia Studies to Evaluate Resistance (TASER) is an initiative of TREAT Asia, a programme of amfAR, The Foundation for AIDS Research, with support from the Ministry of Foreign Affairs of The Netherlands through a partnership with Stichting Aids Fonds (grant number 12454), and the U.S. National Institutes of Health’s National Institute of Allergy and Infectious Diseases, Eunice Kennedy Shriver National Institute of Child Health and Human Development, and National Cancer Institute, as part of the International Epidemiology Databases to Evaluate AIDS (IeDEA; U01AI069907). Queen Elizabeth Hospital and the Integrated Treatment Centre were supported by the Hong Kong SAR Council for AIDS Trust Fund. The Kirby Institute is funded by the Australian Government Department of Health and Ageing, and is affiliated with the Faculty of Medicine, UNSW Australia.

## PASER-M collaborators

Lusaka Trust Hospital (M Siwale), Coptic Hospital (M Labib), KARA Clinic and Laboratory (J Menke), Lusaka, Zambia; Muelmed Hospital, Pretoria, South Africa (M E Botes); Themba Lethu Clinic, Clinical HIV Research Unit (P Ive, and I Sanne) and Department of Molecular Medicine and Haematology (E Letsoalo, WS Stevens, K Steegen), University of the Witwatersrand, Johannesburg, South Africa; Acts Clinic, White River, South Africa (M Hardman); Newlands Clinic, Harare, Zimbabwe (M Wellington, R Luthy); Coast Province General Hospital, International Centre for Reproductive Health Kenya, Mombasa, Kenya (K Mandaliya); Mater Misericordiae Hospital, Nairobi, Kenya (M Dolan); Joint Clinical Research Centre, Fort Portal, Mbale and Kampala, Uganda (C Kityo, S Balinda, W Namala, H Namata, F Senono, R Nakanjako, M Mutebi, I Nankya, P Mugyenyi); Lagos University Teaching Hospital, Lagos, Nigeria (A Osibogun, S Akanmu, T Adeyemo, T Rodoye, H Adelabu); Institute of Human Virology Nigeria, Abuja, Nigeria (N Ndembi); Department of Virology, University Medical Center, Utrecht, The Netherlands (R Schuurman); Amsterdam Institute for Global Health and Development, Kampala, Uganda (C Nalubwama, H Kakooza, M Nakitto, M O’Mello); Department of Global Health, Academic Medical Center of the University of Amsterdam, Amsterdam Institute for Global Health and Development, Amsterdam, the Netherlands (RL Hamers, KCE Sigaloff, TS Boender, P Ondoa, C Manting-de Vries, N Pakker, B Prins, R Meester, FW Wit, JM Lange, TF Rinke de Wit).

## TASER-M collaborators

Queen Elizabeth Hospital (PCK Li, MP Lee), Integrated Treatment Centre (KH Wong), Hong Kong SAR, China; Chennai Antiviral Research and Treatment Clinical Research Site (CART CRS), YRGCARE Medical Centre, VHS, Chennai, India (N Kumarasamy, S Saghayam); Faculty of Medicine, Udayana University & Sanglah Hospital, Bali, Indonesia (TP Merati, F Yuliana); Hospital Sungai Buloh, Sungai Buloh, Malaysia (CKC Lee, BLH Sim); University of Malaya, Kuala Lumpur, Malaysia (A Kamarulzaman, LY Ong); Hospital Raja Perempuan Zainab II, Kota Bharu, Malaysia (M Mustafa, N Nordin); Research Institute for Tropical Medicine, Manila, Philippines (R Ditangco, RO Bantique); Kaohsiung Medical University, Kaohsiung City, Taiwan (YMA Chen, YT Lin); HIV-NAT/Thai Red Cross AIDS Research Centre (P Phanuphak, S Sirivichayakul), Faculty of Medicine Ramathibodi Hospital, Mahidol University (S Sungkanuparph, S Kiertiburanakul, L Chumla, N Sanmeema), Faculty of Medicine Siriraj Hospital, Mahidol University (W Ratanasuwan, R Sriondee), Bangkok, Thailand; Research Institute for Health Sciences, Chiang Mai University, Chiang Mai, Thailand (T Sirisanthana, J Praparattanapan); Chiangrai Prachanukroh Hospital, Chiang Rai, Thailand (P Kantipong, P Kambua); Brown University, Rhode Island, U.S.A. (R Kantor); TREAT Asia, amfAR – The Foundation for AIDS Research (AH Sohn, N Durier, T Singtoroj); The Kirby Institute, UNSW Australia, Sydney, Australia (DA Cooper, MG Law, A Jiamsakul, DC Boettiger).

## Notes

To access the supplementary material to this article please see Supplementary Files under Article Tools online.

## References

[1] UNAIDS Global AIDS update. Vol. 2016, Geneva: UNAIDS; 2016.

[2] Gardner EM, Burman WJ, Steiner JF, Anderson PL, Bangsberg DR Antiretroviral medication adherence and the development of class-specific antiretroviral resistance. Aids. 2009 6 1;23(9):1035–10. cited. 19381075 10.1097/QAD.0b013e32832ba8ec PMC2704206

[3] Bangsberg DR, Perry S, Charlebois ED, Clark RA, Robertston M, Zolopa AR, et al Non-adherence to highly active antiretroviral therapy predicts progression to AIDS. Aids. 2001;15:1181–3. 11416722 10.1097/00002030-200106150-00015

[4] Langebeek N, Gisolf EH, Reiss P, Vervoort SC, Hafsteinsdóttir TB, Richter C, et al Predictors and correlates of adherence to combination antiretroviral therapy (ART) for chronic HIV infection: a meta-analysis. BMC Medicine. 2014;12:142. 25145556 10.1186/s12916-014-0142-1 PMC4148019

[5] Mills EJ, Nachega JB, Bangsberg DR, Singh S, Rachlis B, Wu P, et al Adherence to HAART: a systematic review of developed and developing nation patient-reported barriers and facilitators. Plos Medicine. 2006;2006:e438. 10.1371/journal.pmed.0030438 PMC1637123 17121449

[6] UNAIDS. 90-90-90 An ambitious treatment target to help end the AIDS epidemic 2014. Geneva: UNAIDS; 2014.

[7] O’Connor JL, Gardner EM, Mannheimer SB, Lifson AR, Esser S, Telzak EE, et al Factors associated with adherence amongst 5295 people receiving antiretroviral therapy as part of an international trial. J Infect Dis. 2013;208:40–9. 23204161 10.1093/infdis/jis731 PMC3666133

[8] Mills EJ, Nachega JB, Buchan I, Orbinski J, Attaran A, Singh S, et al Adherence to antiretroviral therapy in sub-Saharan Africa and North America: a meta-analysis. Jama. 2006;296:679–90. 16896111 10.1001/jama.296.6.679

[9] Ortego C, Huedo-Medina TB, Llorca J, Sevilla L, Santos P, Rodríguez E, et al Adherence to highly active antiretroviral therapy (HAART): a meta-analysis. AIDS Behav. 2011;15:1381–96. 21468660 10.1007/s10461-011-9942-x

[10] Safren SA, Biello KB, Smeaton L, Mimiaga MJ, Walawander A, Lama JR, et al Psychosocial predictors of non-adherence and treatment failure in a large scale multi-national trial of antiretroviral therapy for HIV: data from the ACTG A5175/PEARLS Trial. PLoS One. 2014;9:e104178. 25153084 10.1371/journal.pone.0104178 PMC4143224

[11] Hamers RL, Oyomopito R, Kityo C, Phanuphak P, Siwale M, Sungkanuparph S, et al Cohort profile: the PharmAccess African (PASER-M) and the TREAT Asia (TASER-M) monitoring studies to evaluate resistance–HIV drug resistance in sub-Saharan Africa and the Asia-Pacific. Int J Epidemiol. 2012;41:43–54. 21071386 10.1093/ije/dyq192 PMC3304520

[12] WHO Antiretroviral therapy for HIV infection in adults and adolescents: recommendations for a public health approach. Vol. 2006, Geneva: WHO; 2006 revision. 23741771

[13] Oyugi JH, Byakika-Tusiime J, Charlebois ED, Kityo C, Mugerwa R, Mugyenyi P, et al Multiple validated measures of adherence indicate high levels of adherence to generic HIV antiretroviral therapy in a resource-limited setting. J Acquir Immune Defic Syndr. 2004;36:1100–2. 15247564 10.1097/00126334-200408150-00014

[14] Finitsis DJ, Pellowski JA, Huedo-Medina TB, Fox MC, Kalichman SC. Visual analogue scale (VAS) measurement of antiretroviral adherence in people living with HIV (PLWH): a meta-analysis. J Behav Med. 2016;39:1043–55. 27481102 10.1007/s10865-016-9770-6

[15] Paterson DL, Swindells S, Mohr J, Brester M, Vergis EN Adherence to protease inhibitor therapy and outcomes in patients with HIV infection. Ann Intern Med. 2000;133:21–30. 10877736 10.7326/0003-4819-133-1-200007040-00004

[16] Hamers RL, Schuurman R, Sigaloff KCE, Wallis CL, Kityo C, Siwale M, et al Effect of pretreatment HIV-1 drug resistance on immunological, virological, and drug-resistance outcomes of first-line antiretroviral treatment in sub-Saharan Africa: a multicentre cohort study. Lancet Infect Dis. 2012;12:307–17. 22036233 10.1016/S1473-3099(11)70255-9

[17] Nieuwkerk PT, Oort FJ Self-reported adherence to antiretroviral therapy for HIV-1 infection and virologic treatment response: a meta-analysis. J Acquir Immune Defic Syndr. 2005;38(4):445–8. 15764962 10.1097/01.qai.0000147522.34369.12

[18] Liang K-Y, Zeger SL Longitudinal data analysis using generalized linear models. Biometrika. 1986;73:13–22.

[19] The World Bank [Internet] Washington: World Bank Group [cited 2015 5 31]. Available from: http://data.worldbank.org/about/country-and-lending-groups.

[20] Deschamps AE, De Geest S, Vandamme AM, Bobbaers H, Peetermans WE, Van Wijngaerden E Diagnostic value of different adherence measures using electronic monitoring and virologic failure as reference standards. AIDS Patient Care STDS. 2008;22:735–43. 18754705 10.1089/apc.2007.0229

[21] Chaiyachati K, Hirschhorn LR, Tanser F, Newell ML, Bärnighausen T Validating five questions of antiretroviral nonadherence in a public-sector treatment program in rural South Africa. AIDS Patient Care. STDS. 2011;25:163–70. 10.1089/apc.2010.0257 PMC3048836 21269131

[22] WHO Consolidated guidelines on the use of antiretroviral drugs for treating and preventing HIV infection. Recommendations for a public health approach. Second ed. Geneva: WHO; 2016 p. 2016. 27466667

[23] Delgado J, Heath KV, Yip B, Marion S, Alfonso V, Montaner JS, et al Highly active antiretroviral therapy : physician experience and enhanced adherence to prescription refill. Antivir Ther. 1998;8:471–8. 14640395

[24] Simoni JM, Pearson CR, Pantalone DW, Marks G, Crepaz N Efficacy of interventions in improving highly active antiretroviral therapy adherence and HIV-1 RNA viral load. J Acquir Immune Defic Syndr. 2006;43(Suppl S1):23–35. 17133201 10.1097/01.qai.0000248342.05438.52 PMC4044045

[25] Oyomopito R, Lee MP, Phanuphak P, Lim PL, Ditangco R, Zhou J, et al Measures of site resourcing predict virologic suppression, immunologic response and HIV disease progression following highly active antiretroviral therapy (HAART) in the TREAT Asia HIV Observational Database (TAHOD). HIV Med. 2010;11:519–29. 20345881 10.1111/j.1468-1293.2010.00822.x PMC2914850

[26] Ortego C, Huedo-Medina TB, Santos P, Rodríguez E, Sevilla L, Warren M, et al Sex differences in adherence to highly active antiretroviral therapy: a meta-analysis. AIDS Care. 2012;24:1519–34. 22533692 10.1080/09540121.2012.672722

[27] Smith AD, Tapsoba P, Peshu N, Sanders EJ, Jaffe HW Men who have sex with men and HIV/AIDS in sub-Saharan Africa. The Lancet. 2009;374:416–22. 10.1016/S0140-6736(09)61118-1 19616840

[28] UNAIDS. Global Report UNAIDS report on the global AIDS epidemic. Vol. 2013, Geneva: UNAIDS; 2013.

[29] Cambiano V, Lampe FC, Rodger AJ, Smith CJ, Geretti AM, Lodwick RK, et al Long-term trends in adherence to antiretroviral therapy from start of HAART. Aids. 2010;24:1153–62. 20299959 10.1097/QAD.0b013e32833847af

[30] Carrieri MP, Raffi F, Lewden C, Sobel A, Michelet C, Cailleton V, et al Impact of early versus late adherence to highly active antiretroviral therapy on immuno-virological response: a 3-year follow-up study. Antiviral Therapy. 2003;8:585–94. 14760892 10.1177/135965350300800606

[31] Grimsrud A, Sharp J, Kalombo C, Bekker L, Myer L, Town C, et al Implementation of community-based adherence clubs for stable antiretroviral therapy patients in Cape Town. South Africa J Int AIDS Soc. 2015;18:1–8. 10.7448/IAS.18.1.19984 PMC4444752 26022654

[32] Bärnighausen T, Chaiyachati K, Chimbindi N, Peoples A, Haberer J, Newell ML Interventions to increase antiretroviral adherence in sub-Saharan Africa: a systematic review of evaluation studies. Lancet Infect Dis. 2011;11:942–51. 22030332 10.1016/S1473-3099(11)70181-5 PMC4250825

[33] Mills EJ, Lester R, Thorlund K, Lorenzi M, Muldoon K, Kanters S, et al Interventions to promote adherence to antiretroviral therapy in Africa: a network meta-analysis. Lancet HIV. 2014;1:e104–11. 26424119 10.1016/S2352-3018(14)00003-4 PMC5096455

[34] Bennett DE, Jordan MR, Bertagnolio S, Hong SY, Ravasi G, McMahon JH, et al HIV drug resistance early warning indicators in cohorts of individuals starting antiretroviral therapy between 2004 and 2009: World Health Organization global report from 50 countries. Clin Infect Dis. 2012;54(Suppl 4):S280–9. 22544188 10.1093/cid/cis207 PMC3338314

[35] Nachega JB, Parienti JJ, Uthman OA, Gross R, Dowdy DW, Sax PE, et al Lower pill burden and once-daily dosing antiretroviral treatment regimens for HIV infection: a meta-analysis of randomized controlled trials. Clin Infect Dis. 2014;58:1297–307. 24457345 10.1093/cid/ciu046 PMC3982838

